# Thrombin generation as marker to estimate thrombosis risk in patients with abnormal test results in lupus anticoagulant routine diagnostics

**DOI:** 10.1186/1477-9560-11-24

**Published:** 2013-11-12

**Authors:** Klas Boeer, Leonid Cuznetov, Wolfgang Loesche

**Affiliations:** 1 Institut für Klinische Chemie und Laboratoriumsdiagnostik, Jena University Hospital - Friedrich Schiller University Jena, Erlanger Allee 101, 07747 Jena, Germany; 2 Center for Sepsis Control and Care, Jena University Hospital - Friedrich Schiller University Jena, Jena, Germany

**Keywords:** Thrombin generation, Lupus anticoagulant, Thrombosis risk

## Abstract

**Background:**

Lupus anticoagulant (LA) is known to inhibit thrombin generation although patients have an increased risk to develop thrombosis. We tried to determine whether thrombin generation is altered in plasma samples of patients with abnormal test results in LA routine diagnostics and whether its measurement may improve the risk assessment of thrombosis.

**Methods:**

Samples from 63 patients (39 with abnormal test results; 24 controls) were included in the study. Measurement of diluted Russel’s viper venom time (dRVVT) was part of the initial guideline conform diagnostic procedure for detection of LA. In addition, measurement of anticardiolipin-IgM, -IgG and β2-glycoprotein-I-IgM, -IgG were performed. Thrombin generation was measured using two different phospholipid concentrations in the starting reagent.

**Results:**

Analyzing all samples by logistic regression, thrombin generation after induction with high phospholipid concentrations was the best predictor of thrombosis. After preselection of samples with alterations in dRVVT, specificity of selected thrombin generation derived parameters for the detection of previous thrombosis increased in this subgroup.

**Conclusions:**

In patients with phospholipid-dependent prolongation of dRVVT, thrombin generation is variably inhibited and the degree of inhibition corresponds to the occurrence of previous thrombosis. Measuring thrombin generation in patients with phospholipid-dependent dRVVT prolongation may improve risk assessment of thrombosis.

## Background

Antiphospholipid syndrome (APS) is characterized by the presence of antibodies against a complex of phospholipid-binding protein and phospholipids. These antibodies are primarily directed against the phospholipid-binding protein β2-glycoprotein I (β2-GPI) or cardiolipin (aCL) and can be detected by ELISA. If the antibodies interfere with coagulation assays they are described as lupus anticoagulant (LA). Presence of the β2-GPI dependent LA was associated with a high risk of thrombosis in a recent study [1]. Laboratory diagnosis of LA depends on the confirmation in at least one of two specific tests with different assay principles [2]. Depending on the number and type of assays positive for LA or the titer of LA the risk of developing thrombosis varies [3-5]. Very often, LA testing as part of the APS testing is driven by a thrombotic event and patients receive anticoagulation depending on the positivity in different assays and the titer detected [6,7]. However, despite the high risk, recurrence does not occur in all patients [8,9]. Therefore, a better risk stratification seems to be necessary.

Measurement of endogenous thrombin potential (ETP) by thrombography has been attracting attention throughout the last years. Although the methods lack standardization [10] measurement of ETP has been analyzed in a wide variety of patients. With respect to development of thrombosis ETP has been evaluated for the risk assessment in patients with resistance against activated protein C [11], for the prediction of recurrent venous thromboembolism [12,13], as a screening tool for thrombophilic factors [14] and for the detection of APS [15,16]. In the latter studies the effects on thrombin generation in APS patients were particularly attributed to the action of β2-GPI antibodies.

The hypothesis of the present study was that ETP and parameters derived from the measurement of ETP (TG-derived parameters) are altered in patients with abnormal test results of LA diagnostics. It was further evaluated whether alterations correlate with previous thrombotic events.

## Material and methods

### Sample selection

**Figure 1:**
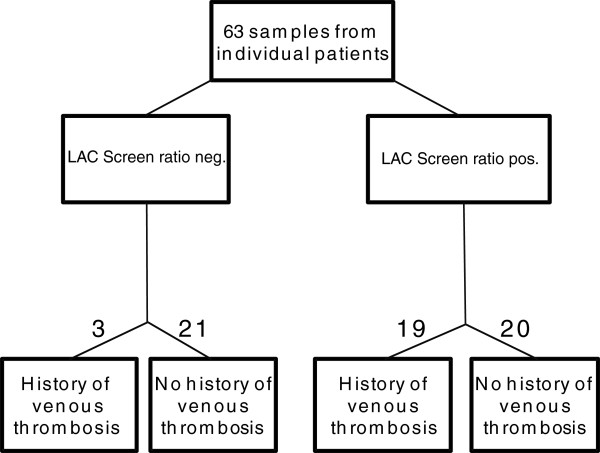
Selection procedure of patients who were included in the study.

Samples from 63 patients were retrospectively selected from the LA testing program of the laboratory from January to December 2009 (Figure 1). Indications for laboratory investigation were previous thrombosis, evaluation of prolonged, activated partial thromboplastin time and clinical history of systemic lupus erythematosus. In case of previous thrombosis samples were collected immediately after the event. Two tests were performed routinely as recommended by established guidelines [17]: (i) diluted Russel’s viper venome time (dRVVT; LAC Screen/LAC Confirm) and (ii) MIXCON-LA. Samples with prothrombin time above reference range were not included because prolonged clotting times may be caused by anticoagulatory drugs. Twenty four negative plasma samples (negative in both screening tests) were randomly selected as controls. All samples with abnormal findings in dRVVT confirmatory testing during the test period were included in the study if the sufficient sample volume and a patient history were available (39 samples). If a patient was repeatedly sampled during the study period only the first sample was included in the study.

Factor-V-Leiden mutation leads to altered thrombin generation only after addition of activated protein C (APC) [14,18] but not in native samples [13]. Therefore, samples from heterozygous Factor-V-Leiden patients were not excluded.

### Laboratory investigation

Samples were centrifuged twice to obtain platelet-free plasma [19] and stored at −80°C. All samples underwent one freeze/thaw cycle (two aliquots, one for the initial diagnostic and one back-up aliquot, were stored). LAC Screen/Confirm (Instrumentation Laboratory, Kirchheim, Germany) were measured according to the instructions of the manufacturer on ACL Top analyzer (Instrumentation Laboratory). Samples were LA-negative if the LAC Screen test result was <45 s. Samples with LAC Screen > 45 s were tested by LAC Screen ratio (LAC Screen_sample_/ LAC Screen _normal plasma_) and were negative if the ratio was < 1.2. Samples with LAC Screen ratio > 1.2 were tested with increased phospholipid concentrations by LAC Confirm. Samples were included if the normalized ratio (LAC Screen_patient_/LAC Screen_normal_ divided by LAC Confirm_patient_/LAC Confirm_normal_) was ≥1.2.

Calibrated automated thrombin generation was measured in platelet-poor plasma using Bio-Tek FLx800 TC fluorometer. Reagents, calibrators and controls were used according to the recommendations of the manufacturer (Tecnoclone, Austria, Vienna). Two assays were performed simultaneously, one with high (RC high, Tecnoclone) one with a low phospholipid concentration (RC low, Tecnoclone) in the starting reagent as described recently [20]. Coagulation was initiated with recombinant human tissue factor. Conversion of substrate was monitored for 60 minutes in 1 minute intervals. Lag time, peak height of thrombin formation, time to peak and area under the curve (ETP) were measured using Bio-Tek KC 4/GEN 5 Software. All samples were analyzed in dublicates. Antithrombin, protein C and protein S were measured using routine methods (HemosIL Liquid Antithrombin; HemosIL Protein C; HemosIL ProS; Instrumentation Laboratory). Factor-V-Leiden and prothrombin G20210A mutation were analyzed by PCR and DNA hybridization (Hain Lifescience; Nehren, Germany). aCL- and β2-GPI IgM/IgG were measured using the Algeria® analyzer (Organtec, Mainz, Germany).

### Statistical analysis

Imprecision was measured by repeated analysis (N = 16) of one quality control sample. Calculations were performed using Analyse-It (Analyse-it Software, Ltd.; Leeds, UK), Excel (Microsoft, Unterschleissheim, Germany) and SPSS 19 (IBM, New York, United States). Comparisons between groups were performed by Mann–Whitney-Wilcoxon-test. Information on the history of the patients was obtained from the hospital information system. The study was approved by the local ethics committee.

## Results

### Patients

Characteristics of patients included in the study are summarized in Table 1. Within the cohort only venous but no arterial thrombotic events occurred. All patients with previous thrombotic events were considered as positive for further analysis regardless whether one or more thrombotic events were recorded in the patient’s history.

**Table 1:** Characteristics of patients included in study

**Female: male (absolute numbers)**	**38:25**
number of patients with history of thrombosis	22
**Location of thrombosis**	
lower extremity and pelvic veins	12
cerebral veins	3
pulmonary embolism	7
**Number of patients tested/number of patients positive for:**	
protein S deficiency	36/0
protein C deficiency	36/0
antithrombin deficiency	45/0
factor-V-Leiden mutation	37/6 heterozygous
prothrombin mutation	37/0

### Precision of the assay

Total imprecision for RC-low reagent were 11.7%, 14.6%, 12.9%, 8.1% and for RC-high reagent 11.7%, 11.5%, 10.8%, 6.0% with respect to lag time, time to peak, peak height of thrombin formation and AUC.

### Influence of β2-GPI and aCL antibodies on thrombin generation

Comparison of TG-derived parameters between the group with (N = 11) and the group without β2-GPI IgM- and/or IgG-antibodies (N = 52) and between the group with (N = 19) and without aCL IgM- and/or IgG-antibodies (N = 44) revealed no significant differences. (p > 0.05). Therefore, positivity for β2-GPI and aCL antibodies was not considered for further analysis.

### Forward stepwise logistic regression analysis for prediction of thrombosis

All parameters (LAC Screen ratio, RC low and RC high: lag time, thrombin peak concentration, time to peak and AUC of thrombin generation) were included in a forward stepwise logistic regression model to identify independent predictors of thrombosis. Using this model variables were selected in the order in which they maximize the statistically significant contribution to the model. Variables were stepwise included in the order which they contribute to the model and the model is recalculated until only variables with p < 0.05 remain. Of all parameters tested RC-high AUC was the best independent predictor of thrombosis (Table 2).

**Table 2:** Logistic regression analysis of all TG-derived parameters and LAC Screen ratio as independent parameters and history of thrombosis as dependent parameter

	**Regression coeffizient B**	**Standard error**	**Wald test**	**p**	**Exp (B)**
RC-high AUC	−0.001	0.000	9.434 (df = 1)	0.002	0.999
Constant	1.653	0.831	3.953 (df = 1)	0.047	5.223

Analysis was performed by forward inclusion. Variables were stepwise included if p – value was < 0.05 and the model is recalculated until only variables with p < 0.05 remain in the model.

### Comparison of TG-derived parameters and LAC Screen test result between patients without and with thrombosis

**Figure 2:**
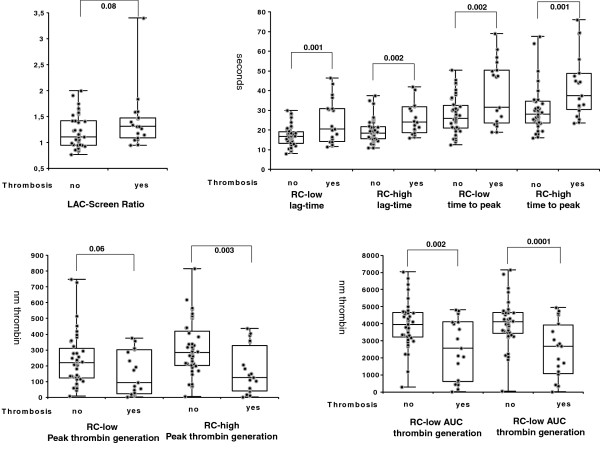
**Box-Whisker- and dot plots of 4 TG-derived parameters (lag time, time to peak, peak thrombin, AUC of thrombin generation) and LAC Screen ratio after induction with 2 different starting reagents (RC-low = low phospholipid concentration, RC-high = high phospholipid concentration).** Significance level was 0.05.

TG-derived parameters differed significantly (except peak thrombin generation with RC-low reagent) between samples from patients with and without thrombosis (Figure 2) while there was no significant difference in LAC Screen ratio. Further analysis of the dot plots revealed a wide spreading of the results most noticeably for the TG-derived parameters time to peak and AUC. Therefore, samples were further subdivided into LAC Screen ratio negative (< 1.2) and positive (> =1.2) samples with and without history of thrombosis.

### Comparison of TG-derived parameters between LAC Screen negative/positive patients without and with thrombosis

**Figure 3:**
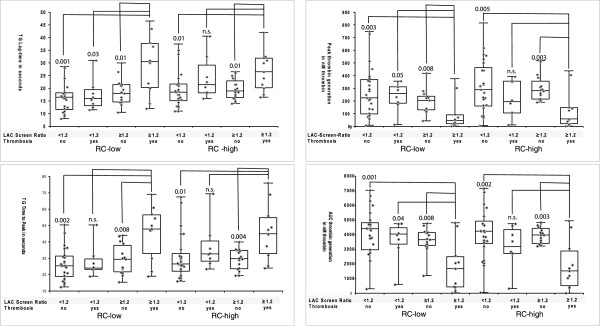
**Box-Whisker- and dot plots of 4 TG-derived parameters (lag time, time to peak, peak thrombin, AUC of thrombin generation) after induction with 2 different starting reagents (RC-low = low phospholipid concentration, RC-high = high phospholipid concentration).** Patients are sorted according to the result of the LAC Screen ratio and clinical history of thrombosis. Results were compared by Mann–Whitney-Wilcoxon test. If not indicated otherwise comparison was statistically non-significant. Significance level was 0.05.

Comparison between the subgroups revealed that TG-derived parameters after induction with RC low reagent differed significantly between samples from patients with history of thrombosis/positive LAC Screen ratio and the other samples (Figure 3). After induction with RC high reagent significant differences were only noticed between the thrombosis negative patients (independent of LAC Screen ratio) and patients with thrombosis/positive LAC Screen ratio (Figure 3).

### ROC-analysis of TG-derived parameters for the prediction of previous thrombosis

Performing ROC (receiver operating characteristics)-analysis of all TG-derived parameters on preselected, LAC Screen ratio positive samples (N = 29; 13 thrombosis, 16 non-thrombosis) revealed high AUC (area under the curve) for all parameters with respect to previous thrombosis (Table 3). ROC-analysis was also performed for the samples with a negative LAC Screen ratio (N = 34; 9 thrombosis, 25 non-thrombosis). However, in this group most TG-derived parameters were not or less pronounced discriminatory between the thrombosis-negative and thrombosis-positive patients (Table 3).

**Table 3:** ROC-analysis of TG-derived parameters for the detection of previous thrombosis in samples from patients preselected by LAC Screen ratio (LAC screen ratio >= 1.2 and LAC screen ratio < 1.2)

**TG-derived parameter**	**ROC (95% CI) (LAC-S ratio >=1.2) N = 29**	**Cases excluded**	**ROC (95% CI) (LAC-S ratio <1.2) N = 34**	**Cases excluded**
**dRVVT**	0.46 (0.24 - 0.68)	none	Not calculated	Not determined
**RC-low lag time**	0.76 (0.56 - 0.97)	2 thrombosis 1 non-thrombosis	0.59 (0.36 - 0.82)	1 thrombosis
**RC-low peak height of thrombin formation**	0.80 (0.57 - 1.00)	2 thrombosis 1 non-thrombosis	0.51 (0.30 - 0.72)	1 thrombosis
**RC-low time to peak**	0.80 (0.58 - 1.00)	2 thrombosis 1 non-thrombosis	0.50 (0.27 - 0.73)	1 thrombosis
**RC-low AUC**	0.81 (0.57 - 1.00)	2 thrombosis 1 non-thrombosis	0.57 (0.35 - 0.78)	1 thrombosis
**RC-high lag time**	0.83 (0.68 - 0.98)	2 thrombosis	0.72 (0.53 - 0.91)	1 thrombosis
**RC_high peak height of thrombin formation**	0.82 (0.60 - 1.00)	2 thrombosis	0.61 (0.39 - 0.83)	1 thrombosis
**RC_high time to peak**	0.80 (0.61 - 0.99)	2 thrombosis	0.70 (0.50 - 0.90)	1 thrombosis
**RC-high AUC**	0.83 (0.60 - 1.00)	2 thrombosis	0.67 (0.46.- 0.87)	1 thrombosis

Four samples had to be excluded from analysis because thrombin generation was inhibited and data could not be further analyzed. This occurred in three samples with positive LAC Screen/LAC Confirm ratio but in one case also in the LAC Screen/LAC Confirm ratio negative group. In this sample from a patient with thrombosis the other initially performed screening test MIXCON-LA ratio was altered.

## Discussion

Patients with LA have an increased risk to develop thrombosis. In clinical practice, testing usually follows a thrombotic event and aims to prevent recurrence. In accordance with current guidelines, LA has to be screened for by a combination of two assays on two different occasions. Depending on the assays used different predictive values have been described for the detection of patients with previous thrombosis [1,21]. However, stratification according to risk of thrombosis or recurrence is a prerequisite to selectively provide those patients with antithrombotic medication who benefit. The measurement of TG-derived parameters in addition to other assays might increase diagnostic specificity.

In general, prolongation of time to peak and lag time on one hand and decrease of peak height of thrombin formation and AUC on the other hand were observed predominantly in samples in which a phospholipid-dependency of the coagulation inhibitor had already been proven before. This has already been shown for LA in the past [15,16]. Association between of β2-GPI and aCL antibodies and prolongation of lag time/reduced thrombin generation is controversial [15,16,22,23]. In our present study the number of antibody-positive patients was small and no significant alteration in any of the TG-derived parameters was observed. Recently Ninnivaggi et al. [23] described that preincubation with phospholipids was necessary to induce a conformational change of β2-GPI resulting in alterations of thrombin generation. Therefore, different testing procedures and the lack of positivity for β2-GPI antibodies in LA-positive patients which has been attributed to the insufficient sensitivity of currently available test kits [15] might have caused the differing results. Because analysis of the effects of β2-GPI antibody positivity was not the main focus of this study this aspect could not be studied further.

When comparing TG-derived parameters between patient with and without thrombosis a relatively wide spreading of the results was detected. Therefore, samples where subdivided further on the basis of the LAC Screen ratio result. This resulted in a more homogeneous distribution of the results. Most notably samples from patients with a positive LAC Screen ratio and a history of thrombosis differed significantly from samples of other patients.

To further analyze this relationship ROC-analysis on these preselected samples was performed to test whether use of TG-derived parameters increased specificity for the identification of patients with previous thrombosis. This resulted in high AUC for all parameters analyzed. It is noteworthy that samples from four patients had to be excluded from analysis because thrombin generation was inhibited and did not provide a reliable result. Among those were two patients with a history of thrombosis. Thus, one could speculate that inhibition of thrombin generation is highly associated with a history of thrombosis.

Acquired resistance against APC has been suggested as a mechanism of LA to induce thrombosis [24-26]. In hereditary APC resistance (factor V Leiden mutation) thrombin generation is not altered [13] and APC has to be added to observe effects on thrombin generation assays [14,18]. However, in acquired APC resistance prolongation of lag time and reduced thrombin generation [27] were described without adding APC. Based on these data one could hypothesize that acquired LA-induced APC resistance is one important underlying mechanism of LA-associated thrombosis as it has already been hypothesized in a recent publication [28]. If patients had a higher thrombotic risk, testing by thrombography or perhaps also for acquired APC resistance supplementing the established work-up could result in different stratification with respect to medication and improve the outcome of patients.

The most serious limitation is the retrospective character of the study. In other studies a thrombogenic state has been associated with increased thrombin generation [12,13]. In our study decreased thrombin generation was noticed and this was predominantly observed in samples with positive dRVVT. In most of these samples a yet unconfirmed LA might have been present because LA has also been shown to inhibit thrombin generation [15,16]. However, the risk of previous thrombosis does not necessarily reflect the risk of future thrombosis. This aspect can only be addressed by a prospective study in a larger patient cohort. The latter approach could also avoid a second shortcoming of the study because not all but only randomly selected LA-negative samples from the study period were included as controls. Therefore, positive samples were overrepresented which might have biased the analysis.

Another limitation of the study was that only samples with abnormal initial test results and not diagnostically proven LA-positive samples were included. In some patients coagulation assays may have normalized within the following 12 weeks. However, this does not necessarily affect the interpretation of the results because the analysis was not based on the diagnosis LA (“yes” or “no”) but on the initial, quantitative changes in coagulation assays which are most likely associated with the titer of a coagulation inhibitor the latter being in most cases LA.

## Conclusions

TG-generation is reduced in samples from patients with alterations detected by dRVVT hinting towards the presence of LA. In addition, presence of reduced thrombin generation in these samples was associated with a history of thrombosis. Therefore, TG should be evaluated in a larger patient collective with respect to it diagnostic role when testing for LA.

## Abbreviations

LA: Lupus anticoagulant; dRVVT: diluted Russel’s viper venom time; APS: Antiphospholipid syndrome; ETP: Endogenous thrombin potential; APC: Activated protein C; SCT: Silica clotting time; KCT: Kaolin clotting time; β2-GPI-IgG, IgM: β2-glycoprotein-I-IgM, -IgG; aCL-IgG, IgM: Anti-cardiolipin-IgG, IgM; ROC: Receiver operating characteristics; AUC: Area under the curve.

## Competing interests

This research received no specific grant from any funding agency in the public, commercial, or not-for-profit sectors.

## Authors’ contributions

KB conceived and designed the study; carried out experiments; performed parts of the statistical analysis; helped to draft the manuscript LC carried out experiments; performed parts of the statistical analysis; helped to draft the manuscript WL participated in the design and coordination of the study and helped to draft the manuscript. All authors read and approved the final manuscript.
